# Supplementary figures and images for: Genome-Wide Identification of ATP-Binding Cassette (ABC) Transporter Gene Family and Their Expression Analysis in Response to Anthocyanin Transportation in the Fruit Peel of Eggplant (Solanum melongena L.)
Source: Int J Mol Sci. 2025 Aug 14;26(16):7848. doi: 10.3390/ijms26167848 (PMC12387006; doi:10.3390/ijms26167848)

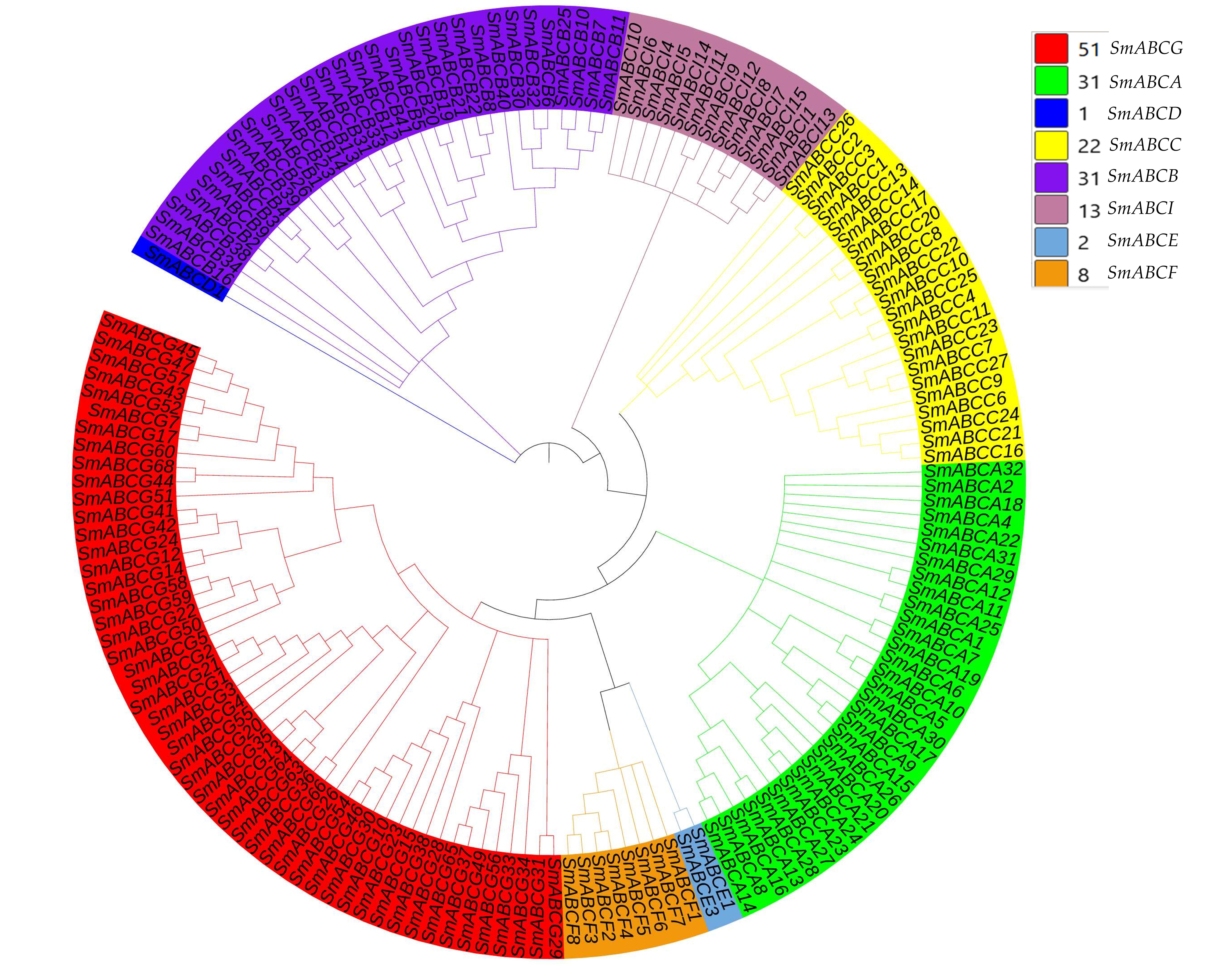

Supplement: Supplementary file 1 [file ijms-26-07848-s001.zip › Figure S1. Phylogenetic tree of SmABC genes in eggplant .jpg]

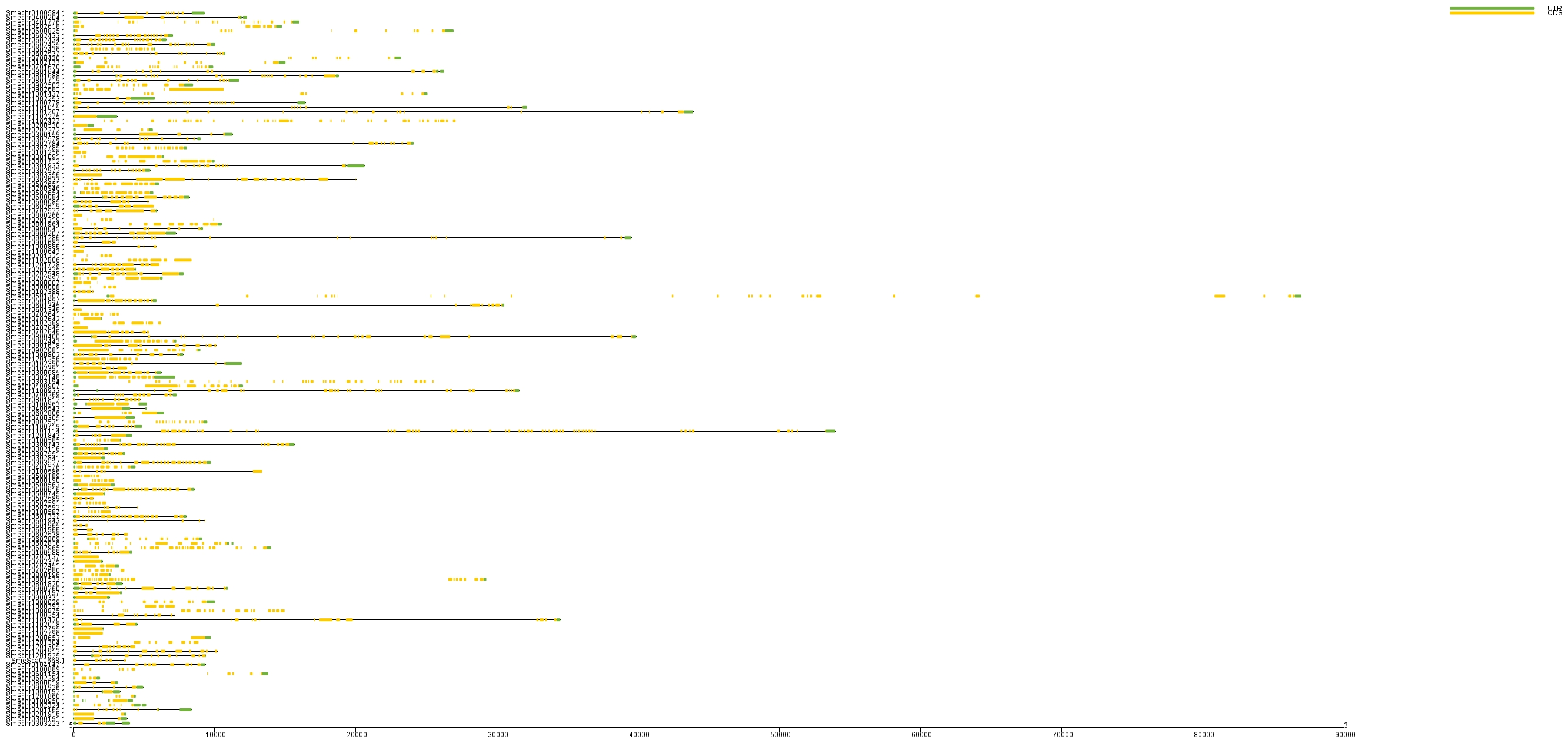

Supplement: Supplementary file 1 [file ijms-26-07848-s001.zip › Figure S2. Gene structure of SmABC in eggplant.jpg]

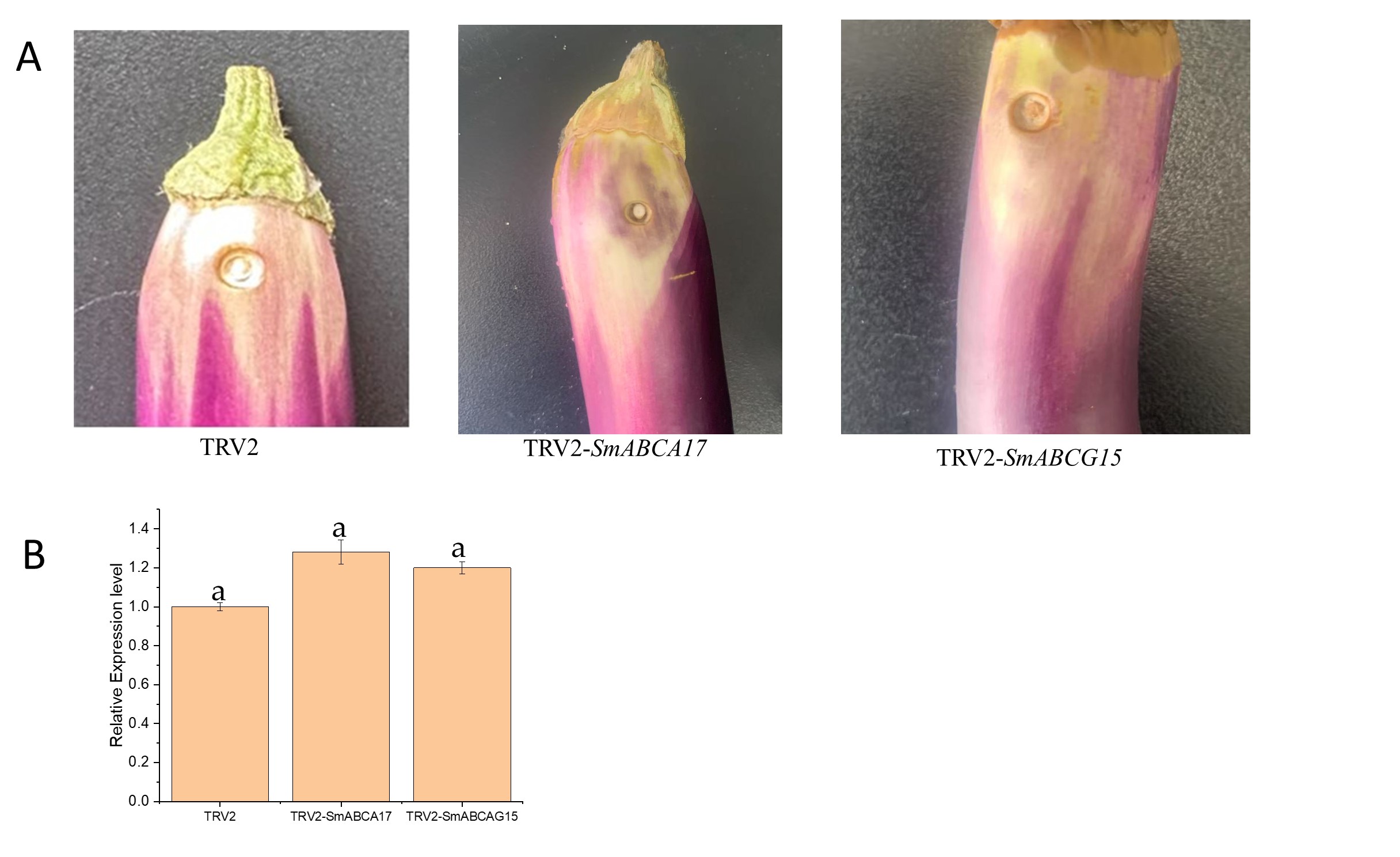

Supplement: Supplementary file 1 [file ijms-26-07848-s001.zip › Figure S3. VIGS of SmABCA17 and SmABCG15..jpg]
